# *C. elegans* Spastin/*spas-1* Is Required for Axon Regeneration and Maintenance

**DOI:** 10.1523/ENEURO.0275-25.2025

**Published:** 2026-02-03

**Authors:** Mary Claire Howell, Rachid Michel El Bejjani

**Affiliations:** Biology Department, Davidson College, Davidson, North Carolina 28035

**Keywords:** *C. elegans*, maintenance, regeneration, spastin

## Abstract

Spastin is a conserved microtubule-severing enzyme mutated in hereditary spastic paraplegia. The role that spastin plays in the cell biology of axon regeneration and degeneration has recently been investigated in *Drosophila*. We show that the *C. elegans* spastin ortholog, *spas-1*, is expressed in GABA motor neurons, in addition to the known expression in touch receptor neurons (TRNs) and that it is required for axon regeneration in the GABA motor neurons after in vivo laser axotomy. We identified no neuronal developmental defects in the GABA motor neurons and only minor branching variations in the TRNs. However, we show that *spas-1* is required for the long-term maintenance of TRN axons in *C. elegans*, as older *spas-1* null *C. elegans* show a significant increase in specific axonal morphological defects compared with the wild type as identified by confocal microscopy in aged animals. Together, our results suggest that spastin is required for regrowth and maintenance of axons in *C. elegans*, consistent with previous reports in *Drosophila*.

## Significance Statement

Spastin is a conserved microtubule-severing enzyme that is mutated in hereditary spastic paraplegia. We show that, like in other systems, *C. elegans* spastin is expressed in motor and sensory neurons and that it is required for axon regeneration and maintenance. These data confirm findings in other systems and expand knowledge of the role of spastin in the fundamental cell biology of axons during regeneration and long-term maintenance.

## Introduction

Axons must maintain their elongated structure over the lifespan of an animal to mediate nervous system function. Physical trauma or disease can lead to the severing of axons or to their progressive degeneration, causing paralysis and other functional defects. Several structural components of invertebrate and vertebrate neurons are known to mediate resistance to mechanical stress (Hammarlund et al., 2007; Leterrier, 2024; Pan et al., 2024). Microtubules are essential components of the structure, function, and intracellular transport of cells in general and neurons in particular, and mutations that affect microtubule (MT) structure and function are associated with developmental defects and neurodegeneration (Lasser et al., 2018; Sferra et al., 2020).

Spastin is a highly conserved MT-severing enzyme mutated in spastic paraplegia patients (Errico et al., 2002). By severing microtubules, spastin regulates diverse cellular processes, including transport, endosome, lysosome, and autosome fusion and fission, cytokinesis, and ER homeostasis (Liu et al., 2021; Costa and Sousa, 2022). Over 150 mutations in the SPAST gene are linked to hereditary spastic paraplegia (HSP), a human neurodegenerative disease marked by progressive limb weakness and spasticity due to defects in the long axonal tracts of motor neurons (Blackstone, 2012). The role of spastin as a MT-severing enzyme that is conserved across taxa led to several investigations of the mechanism of function of the enzyme in invertebrate genetic models, which are amenable to genetic analysis and mechanistic, cell biological investigations in vivo (McNally and Roll-Mecak, 2018).

The initial genetic analysis studies of spastin function in invertebrates used the *Drosophila* system. Spastin mutant flies display severe motor impairments that are rescued by the expression of human or *Drosophila* spastin, confirming structural and functional conservation of spastin in this system (Kammermeier et al., 2003; Sherwood et al., 2004; Du et al., 2010). Further studies in *Drosophila* described the MT-severing function of the protein (Roll-Mecak and Vale, 2005). Importantly, more recent studies using in vivo laser axotomy in fly larvae showed that spastin is required for axon regeneration (Stone et al., 2012; Rao et al., 2016).

Spastin's MT-severing function and functional domains are also conserved in *C. elegans* (Matsushita-Ishiodori et al., 2007). Through severing microtubules, *spas-1*, the *C. elegans* ortholog of spastin, regulates diverse processes, including motor neuron synapse remodeling and lipid droplet metabolism (Kurup et al., 2015; Papadopoulos et al., 2015). Additionally, like human spastin, *C. elegans spas-1* is expressed in the nervous system, specifically in the ventral nerve cord, nerve ring, and touch receptor neurons (TRNs; Brown and El Bejjani, 2017).

Because of the availability of neuronal specific promoters and powerful genetic tools, the worm's short lifespan, transparent body, and efficient laser axotomy and imaging techniques in multiple larval and adult stages and in many types of neurons, *C. elegans* is an exceptionally well-suited model to study the genetic and cell biological mechanisms of axon regeneration and neurodegeneration (El Bejjani and Hammarlund, 2012a; Hayden et al., 2022; Torres et al., 2025). Despite the conservation of spastin's MT-severing function, its expression in multiple neuron types, and the amenability of *C. elegans* for neurodegeneration and axon regeneration studies, the roles of spastin in axon regeneration and maintenance in this model are not described. Here we show that the *C. elegans* ortholog of spastin, *spas-1*, is expressed in GABA motor neurons and that it is required for GABA motor neuron axon regeneration. We also describe increased structural defects in long TRN axons in *spas-1* mutants as early as the L4 stage, progressively worsening in older adults, confirming a phenotype observed by others (Lee et al., 2025).

## Materials and Methods

### *C. elegans* strains

Animals were maintained on NGM agar plates with *E. coli* OP50 as a source of food (Stiernagle, 2006). Temperature was controlled at 20°C. Detailed tables including worm strains, genotypes, transgenes, clones, and PCR primers can be found in Table 1.

**Table 1.** Supplemental experimental procedures (primer sequences)

Primer	Sequence
OK1608 genotyping FW	ACTTGAGGAACTCTATCTTTCAAAGC
OK1608 genotyping REV	TTAGCAACCGAAACTTCGAGAG
*p*RB1084FW	ggggacaactttgtatagaaaagttgTTCAAGAAGACAAATTCGGTAAGCC
*p*RB1084REV	ggggactgcttttttgtacaaacttgTGGAACTGAAAATTTAATACAATTGGAAAAC
*p*RB1085FW	ggggacaagtttgtacaaaaaagcaggctaaaATGTTCGCCTTTTCAAAAGGTCC
*p*RB1085REV	ggggaccactttgtacaagaaagctgggtTTAGCAACCGAAACTTCGAGAGA

### Axotomy

All experiments were performed in parallel with a matched control. L4-stage hermaphrodites were mounted in a slurry of 0.05-μm-diameter polystyrene beads (Polysciences) to immobilize the animals. Immobilization with beads does not affect animal survival, and animals begin crawling immediately after recovery. Commissures in the tail region of the animal posterior to the vulva were severed (GABA neurons: VD and DD). Commissures were visualized with a Nikon Eclipse 80i microscope using a 100× Plan ApoVC lens (1.4 NA) and a Hamamatsu Orca camera. Selected axons were cut using a Micropoint laser from Photonic Instruments (10 pulses, 20 Hz). Axotomized animals were recovered on agar plates and remounted 16–18 h later for scoring. At least 30 axons were scored for most genotypes (2–3 cut axons per animal), and strains with controls from the same experimental day were compared. Only axons with a distal stump as evidence of a complete cut were scored. Axons with a visible growth cone that had progressed past the cut site and axons that had regenerated to the dorsal nerve cord were scored as positive. Axons with no growth or with only filopodial extensions and no progression past the cut site were counted as negative. Statistical significance and exact *p* value were calculated using a Fisher's exact test on contingency tables in GraphPad Prism. The exact *p* value for each pairwise comparison is shown above each graph.

### Molecular biology

Plasmids were assembled using Gateway recombination (Invitrogen). Entry clones were generated using Phusion DNA polymerase (Thermo Fisher scientific). Primers, templates, and plasmid names are listed in Tables 1–4. Genotyping PCR was performed using a 2× OneTaq mastermix from New England Biolabs. Primer sequences and construction details are shown in Tables 1 and 2.

**Table 2.** Gateway expression clones

Plasmid	Promoter [4-1]	Entry [1-2]	Terminator [2-3]	Description
*p*RB1086	*p*MH522 [*Punc-47* 4-1]	*p*RB1085	*unc-54* 3’UTR [2-3]	*PGABA::spas-1*
*p*RB1087	*p*RB1084[*Pspas-1* 4-1]	*p*RB1085	*unc-54* 3’UTR [2-3]	*Pspas-1::spas-1*
*p*RB1110	*p*RB1084[*Pspas-1* 4-1]	*mCherry* [*1-2*]	*unc-54* 3’UTR [2-3]	*Pspas-1::mCh*

**Table 3.** Transgene name and concentrations

Transgene	Allele	Content
*PGABA::spas-1*	*axrEx58 and axrEx59*	*Punc-47*::*spas-1* (20 ng/μl); P*myo-2*::GFP (2 ng/μl)
*Pspas-1::mCh*	*axrEx08*	*Pspas-1::mCh* (20 ng/μl); P*myo-2*::GFP (2 ng/μl)
*Pspas-1::spas-1*	*axrEx61 and axrEx62*	*Pspas-1::spas-1* (20 ng/μl); P*myo-2*::GFP (2 ng/μl)

**Table 4.** Worm strains

Strain name	*Genotype*	Description
RB1411	*spas-1(ok1608) V*	Deletion allele, from the CGC
XE1007	*oxIs12[Punc-47::GFP, lin-15+] X*	GABA neurons express GFP
REB127	*spas-1(ok1608) V; oxIs12[Punc-47::GFP, lin-15+] X*	Crossed from RB1411 and XE1007
CZ10175	*zdIs5 [Pmec-4::GFP + lin-15(+)] I*	Mec neurons express GFP
XE1598	*spas-1(ok1608) V; zdIs5 [Pmec-4::GFP + lin-15(+)] I*	Crossed from CZ10175 and RB1411
REB136	*spas-1(ok1608) V; oxIs12[Punc-47::GFP, lin-15+] X; axrEx58[Punc-47::spas-1]*	Transgene expresses *spas-1* in GABA neurons
REB137	*spas-1(ok1608) V; oxIs12[Punc-47::GFP, lin-15+] X; axrEx59[Punc-47::spas-1]*	Transgene expresses *spas-1* in GABA neurons
REB133	*spas-1(ok1608) V; oxIs12[Punc-47::GFP, lin-15+] X; axrEx61[spas-1::spas-1]*	Rescue line
REB134	*spas-1(ok1608) V; oxIs12[Punc-47::GFP, lin-15+] X; axrEx62[spas-1::spas-1]*	Rescue line

### Transgenics

Transgenic animals were obtained by microinjection as described (Mello et al., 1991). Transgene name, content, and concentrations are listed in Supplementary Experimental Procedures. For most strains, stable transgenic lines were selected based on GFP expression in the pharyngeal muscles from a *Pmyo-2*::GFP coinjection marker. Additional details are shown in Tables 3 and 4.

### Regeneration and expression imaging

For expression imaging and for scoring of axon regeneration, animals were immobilized with 20 mM sodium azide on a 3% agarose pad, coverslipped with a #1.5 cover glass, and imaged immediately using an UltraVIEW VoX (PerkinElmer) spinning disc confocal and 60 or 100× CFI Plan Apo, NA 1.0 oil objectives. Images shown are maximum intensity projections of corresponding *z*-stacks.

### Axon maintenance/degeneration scoring

To investigate the role of *spas-1* in TRN axon maintenance over time, we compared aged animals (10 d adults) with L4 animals. *spas-1(ok1608)* and control L4-stage hermaphrodites were picked onto fresh NGM agar plates. Ten-day-old adults, 10 D after the L4 stage, are noted as 10 D adults. To isolate aging animals from their progeny and to prevent nematode starvation, adults on each plate were moved onto a new plate every day for the duration of the experiment.

Control and *spas-1(ok1608)* aged and L4 animals were imaged and assessed for axon defects. Animals were mounted on 2% agarose pads, immobilized with ∼10 μl 0.5 M levamisole and 1% tricaine in M9, secured with a #1.5 cover glass, and imaged immediately using a Nikon Eclipse Ti2 confocal microscope and 10, 20, or 40× objectives. Images shown are maximum intensity projections of corresponding *z*-stacks.

*Z*-stack images were scored to determine the mean number of axon defects per experimental group. To calculate the mean neuron defects per worm, the sum of axon defects defined as the number of neurons that exhibit wavy processes and the number of breaks or soma outgrowth in each neuron was divided by the number of worms in each experimental group as described previously (Toth et al., 2012). The experimenter was blinded to the genotype during all scoring. Statistical significance and *p* value were calculated using a Mann–Whitney test or Fisher's exact test in GraphPad Prism.

## Results

### *spas-1* is expressed in GABA motor neurons

**Figure 1.**
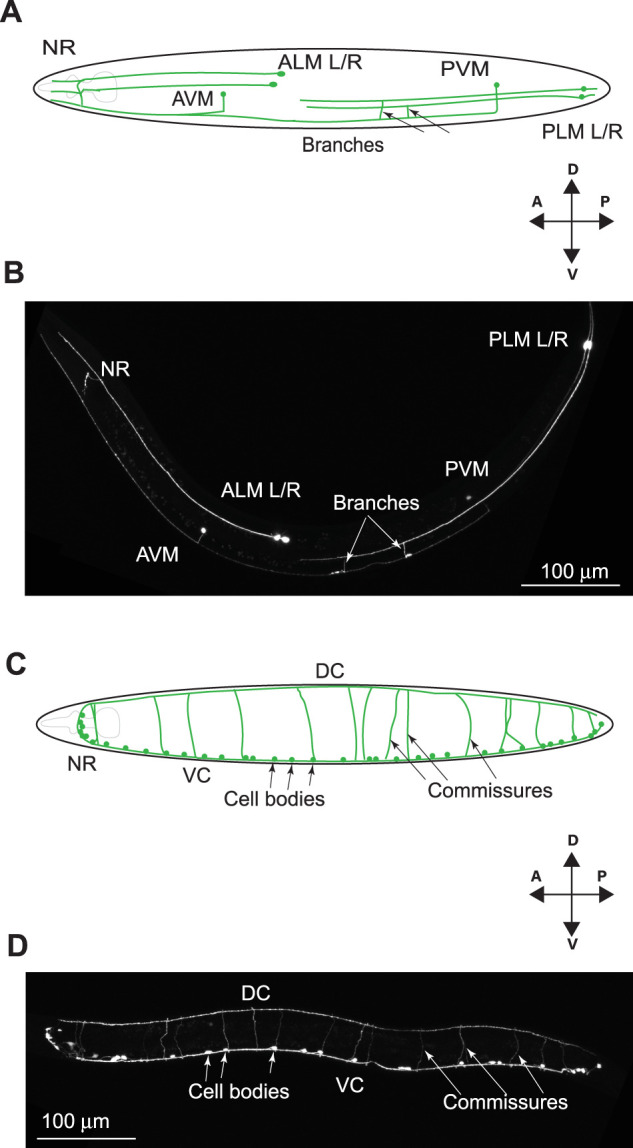
Anatomy of the TRNs and GABA motor neurons in *C. elegans*. ***A***, Cartoon of the TRNs showing the left and right anterior lateral mechanosensory neurons (ALM L/R), left and right posterior lateral mechanosensory neurons (PLM L/R), the posterior ventral mechanosensory neuron (PVM), and the anterior ventral mechanosensory neuron (AVM). ***B***, Stitched, maximum intensity projection of the TRNs in L4-stage hermaphrodite animal imaged at 400× showing all structures cartooned in ***A***. ***C***, Cartoon of the GABA motor neurons showing the ventral and dorsal cords (VC and DC, respectively) and the GABA commissures that extend from the ventral cord to the dorsal cord on the right side of the animal. The nerve ring of the animal is also shown (NR). ***D***, Stitched, maximum intensity projection of the TRNs in L4-stage hermaphrodite animal imaged at 400× showing all structures cartooned in ***C***. Dorsal (D), ventral (V), anterior (A), and posterior (P) orientations are depicted. Scale bar, 100 μm.

**Figure 2.**
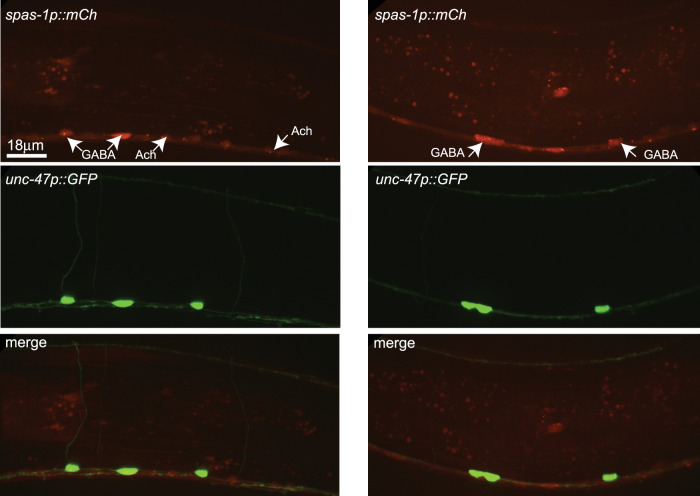
spas-1 is expressed in the GABA motor neurons and other neurons. Top panels, mCherry expression driven by the native *spas-1* promoter previously described in Brown and El Bejjani (2017). Middle panels, GFP expression driven by the GABA motor neuron specific *unc-47* promoter. Bottom panels, Merge of mCh and GFP confirming *spas-1* expression in GABA neurons. Scale bar, 18 μm.

In *C. elegans*, the GABA motor neurons and the TRNs are well-described neuron types that can be identified by expressing soluble GFP in the respective neuron types with tissue-specific promoters (Fig. 1). *spas-1* is expressed in multiple tissues in *C. elegans*, including in the nervous system in general, in the TRNs, and in the intestine (Brown and El Bejjani, 2017). We set out to determine if *spas-1* expression is detectable in GABA neurons. We crossed a transgenic line expressing mCherry driven by the regulatory region 521 bp upstream of the *spas-1* ORF with a strain in which GABA neurons are visualized with GFP using the *unc-47* promoter (a GABA vesicle transporter, PGABA). Two-color confocal imaging of the strains shows mCherry expression in the GABA neurons. Merged images show coexpression in representative images of the same animal (Fig. 2). Our results also show expression in ventral cord neurons other than GABAs, likely cholinergic motor neurons.

### *spas-1* is not required for the development of motor neurons and has minor developmental defects in the TRNs

**Figure 3.**
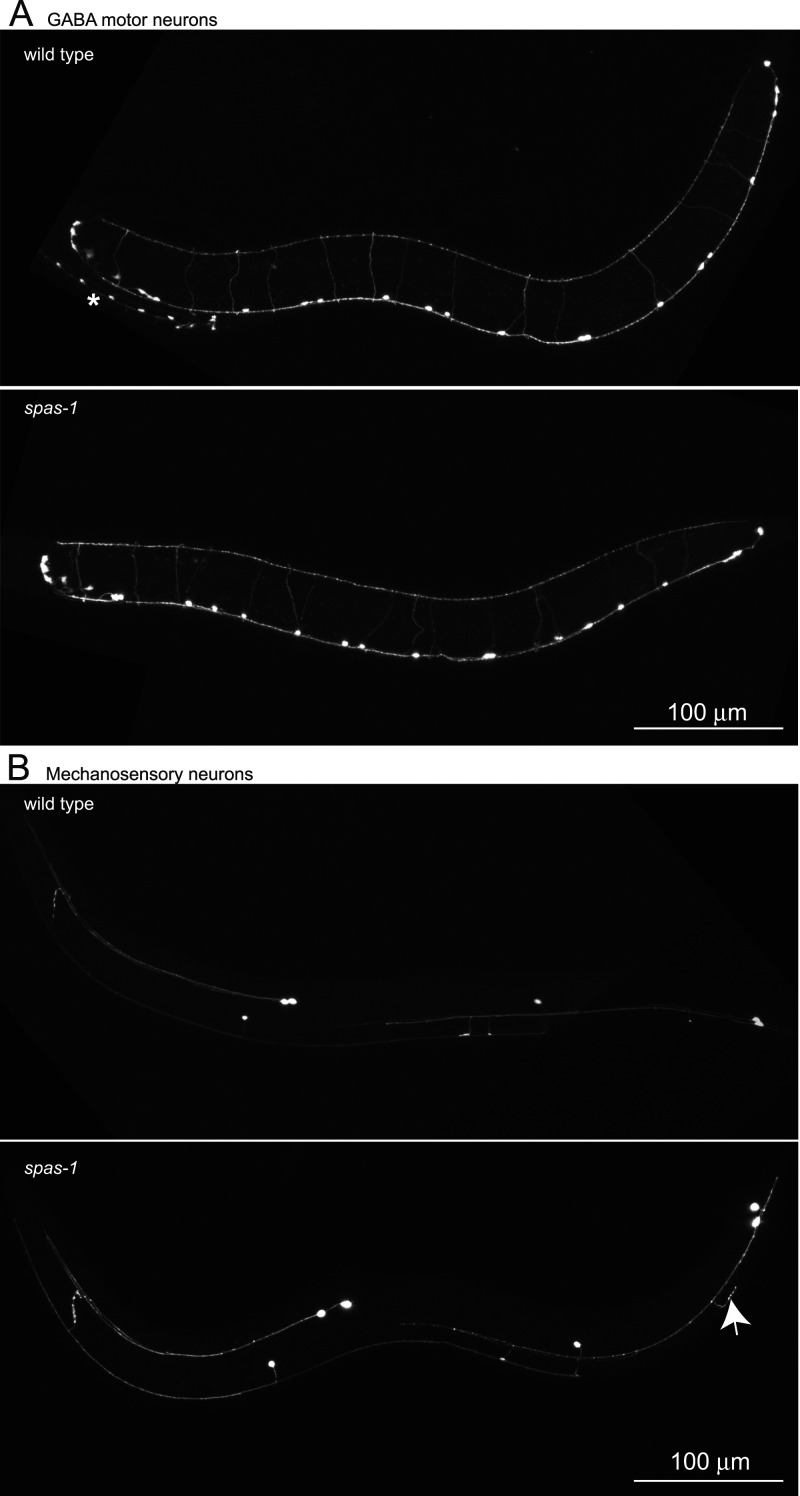
spas-1 is not required for the development of motor neurons and has minor developmental defects in the TRNs. ***A***, Maximum intensity projection of confocal *z*-stack of the worm in *oxIs12 [PGABA::GFP]* (top panel) and *spas-1(ok1608); oxIs12 [PGABA::GFP]* (bottom panel). Asterisk in the top panel shows an L1 animal near the imaged L4. ***B***, Maximum intensity projection of confocal *z*-stack images of the worm in *zdIs5 [Pmec-4::GFP]* (top panel) and *spas-1(ok1608); zdIs5 [Pmec-4::GFP]* (bottom panel). The arrow shows a misplaced branch in the *spas-1* mutant. Scale bar, 100 μm.

Research in the *Drosophila* system described severe locomotion defects in flies lacking the spastin ortholog, suggesting severe defects in neuron development (Kammermeier et al., 2003). Surprisingly, a deletion of the *C. elegans* spastin ortholog, *spas-1*, does not affect normal locomotion in young animals despite a conserved MT-severing function (Matsushita-Ishiodori et al., 2007). We set out to determine if initial neuronal development is affected in *spas-1* deletion mutants in two neuron types. We crossed a *spas-1(ok1608)* deletion allele into a GABA neuron marker (Fig. 3*A*; McIntire et al., 1997) and to a marker for TRNs (TRNs; Fig. 3*B*). We imaged neuron structure in the mutants on the respective backgrounds using confocal microscopy and did not identify any major axon guidance or structural defects, suggesting that *C. elegans spas-1* is not required for initial neural development. Intriguingly, our preliminary observations identified a putative branching variation requiring additional investigation in PLM neurons in some *spas-1(ok1608)* animals regardless of age (Fig. 3*B*, bottom panel, arrow). We feel that, while interesting and worth investigating in more detail, this phenotype lies outside the scope of the paper we present here, and we do not investigate it further at this time.

### *spas-1* is required for axon regeneration in GABA motor neurons

**Figure 4.**
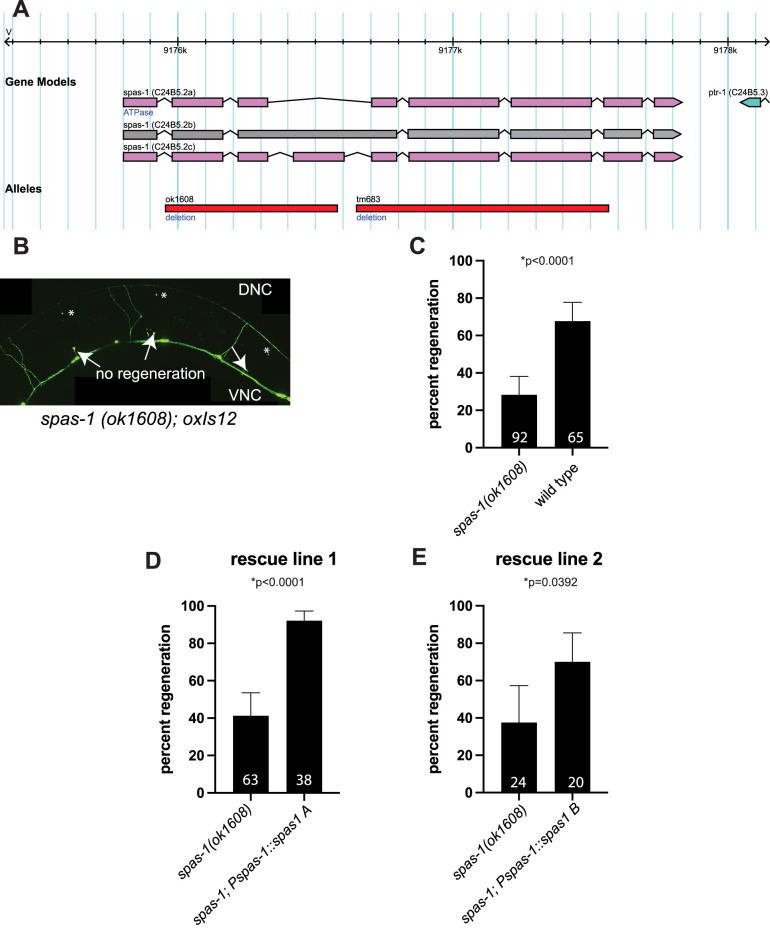
*spas-1* is required for the regeneration of GABA motor neurons after laser axotomy. ***A***, Gene model of *C. elegans spas-1* showing two verified isoforms a and c and one nontranscribed isoform b. *ok1608* deletes all isoforms past the first exon and was used in this study. *tm683* is another deletion that was not used in the study (Sternberg et al., 2024). ***B***, Representative micrograph of a maximum intensity projection of the posterior GABA motor neurons of an L4 hermaphrodite left to recover on NGM agar with *E. coli* for 18 h after laser axotomy. Arrows show proximal axon stumps that failed to regenerate; asterisks show distal stumps where the severed axons were once connected. DNC, dorsal nerve cord; VNC, ventral nerve cord. ***C–E***, Percentage of axons that regenerated past the cut site in the midline or failed to regenerate in wild type, *spas-1(ok1608)*, and two separate extrachromosomal array transgenic lines expressing genomic *spas-1* driven by the native *spas-1* promoter described in Brown and El Bejjani (2017). All axotomies were performed on L4 hermaphrodites and scored 18 h after laser axotomy. The number of scored axons is shown in the bar graph for each genotype. Two-sided Fisher's exact test. Exact *p* values of 2 × 2 contingency tables are noted in each graph; asterisks denote statistically significant result.

Spastin is required for axon regeneration in *Drosophila* (Stone et al., 2012; Rao et al., 2016). *C. elegans* GABA motor neurons are a well-described model for axon regeneration studies (Fig. 1*C*,*D*), and multiple conserved pathways involved in axon regeneration were discovered and further described using genetic screens in spontaneous breaking mutants and in vivo laser axotomy experiments (Hammarlund and Jin, 2014; Byrne and Hammarlund, 2017). Because GABA neurons express spastin, and because *Drosophila* spastin is required for axon regeneration, we genetically crossed the *spas-1(ok1608)* deletion allele (Fig. 4*A*; Sternberg et al., 2024) to the GABA motor neuron marker, *oxIs12*, and performed in vivo laser axotomy. We then quantified the proportion of cut axons that regrew past the cut site at the midline 16–18 h after injury. We used the distal stump of the original connected axon as evidence of successful axotomy (Fig. 4*B*). We show that, similarly to what was previously observed in the fly, *spas-1* is required for axon regeneration in *C. elegans* (Fig. 4*C*). Importantly, expression of wild-type genomic *spas-1* driven by the endogenous promoter previously used for our GABA neuron expression study (Fig. 2) is sufficient to rescue regeneration to rates similar to those seen in wild-type animals (Fig. 4*D*,*E*).

### *spas-1* functions cell autonomously in GABA motor neurons to regulate axon regeneration

**Figure 5.**
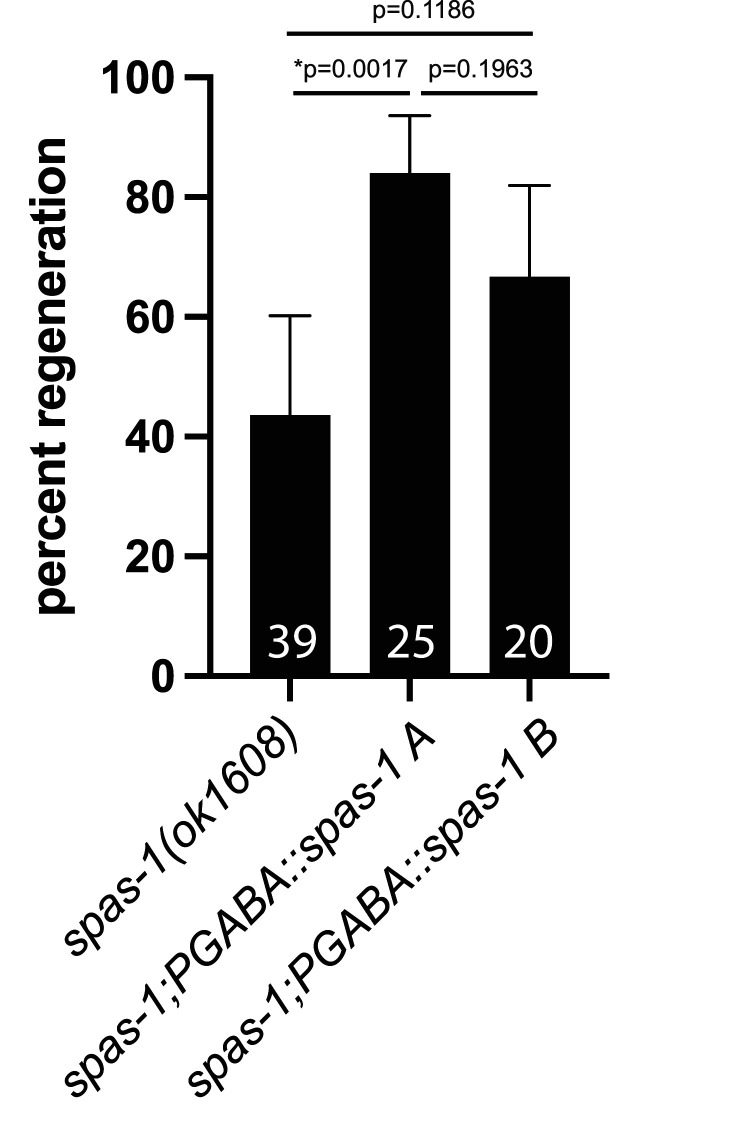
*spas-1* expression in GABA motor neurons is sufficient to rescue regeneration. Genomic *spas-1* driven by the *unc-4*7 GABA motor neuron promoter shows increased regeneration in response to axotomy compared with *spas-1(ok1608)* in two separate extrachromosomal transgenic lines; increased regeneration is statistically significant in Line A. The number of scored axons is shown in the bar graph for each genotype. Two-sided Fisher's exact test. Exact *p* values of 2 × 2 contingency tables are noted in each graph; asterisks denote statistically significant result.

Because *spas-1* is expressed in several neuron types and in other tissues, such as the intestine (Brown and El Bejjani, 2017), we set out to determine if *spas-1* is acting cell autonomously to affect axon regeneration in the GABA neurons. We cloned the *spas-1* ORF under the regulation of the well-described *unc-47* promoter that drives expression in GABA motor neurons specifically (PGABA; McIntire et al., 1997). We show that expression of *PGABA::spas-1* in the *spas-1(ok1608)* mutant is sufficient to rescue axon regeneration rates (Fig. 5), suggesting that *spas-1* functions cell autonomously in GABA motor neurons as a positive regulator of axon regeneration.

### *spas-1* is required for axon maintenance in the TRNs

**Figure 6.**
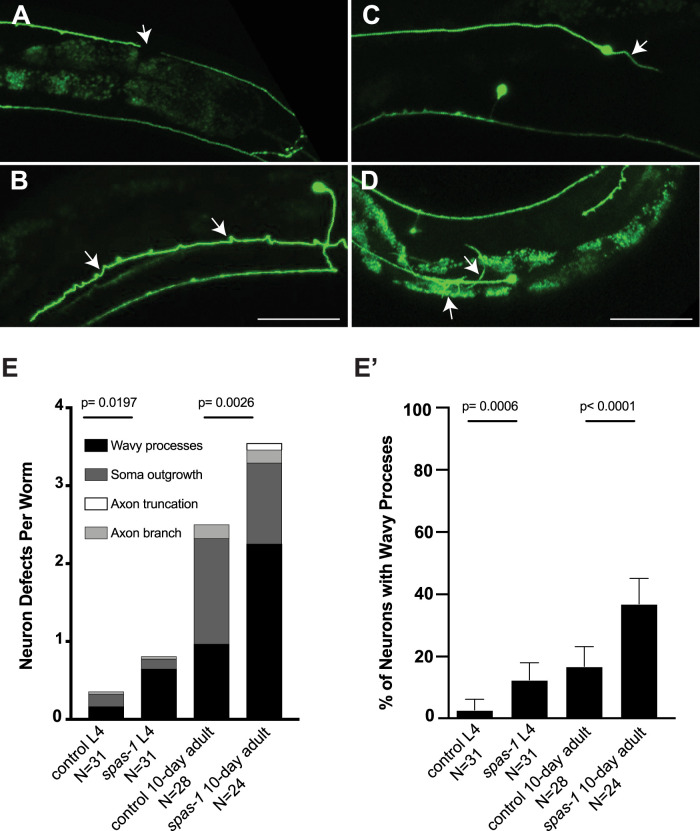
*spas-1* is required for axonal maintenance in the TRNs. ***A–D***, Maximum intensity projections of confocal *z*-stacks of scored defects from representative *spas-1(ok1608)* 10-d-old adult worms. Images show broken axons, arrow (***A***); branching from the soma, arrow (***C***); wavy processes, arrow (***B***); and axonal branching, arrow (***D***). ***D*** also shows wavy processes similar to those shown in ***B***. Scale bar, 100 μm. ***E***, Mean defects per worm encompassing all four defects shown above, as described in the methods. *N* = Numbers of worms scored per condition, Mann–Whitney *U* test calculated *p* values are displayed for two separate comparisons. ***E*’**, The percentage of neurons with wavy processes, replotted from the same experiment as ***E***. Error bars are 95% confidence intervals. Fisher's test calculated exact *p* values are displayed for two separate comparisons.

Because spastin mutations are associated with dominant HSP and because fly mutants display severe motor movements, while *C. elegans spas-1* deletion alleles move indistinguishably from wild-type worms at a young age, we sought to investigate whether *spas-1* is required for *C. elegans* TRN maintenance in older adult *C. elegans* (Brown and El Bejjani et al., 2017; Meyyazhagan and Orlacchio, 2022). We crossed a *spas-1(ok1608)* deletion allele into a TRN marker and imaged L4 and 10 d adults using confocal microscopy. Imaging revealed that *spas-1* is required for axonal maintenance; compared with control animals, *spas-1(ok1608)* animals displayed significantly higher levels of axon structural defects, including breaks (Fig. 6*A*), branching from the soma (Fig. 6*C*), branching from axons (Fig. 6*D*), and wavy processes (Fig. 6*B*). As the animal ages, structural defects increase, indicating the role of *spas-1* in long-term axon maintenance (Fig. 6*E*). Notably, we found that the most pronounced age-dependent defect that is increased in the *spas-1(ok1608)* animals is the appearance of wavy processes in the axon (Fig. 6*B*,*E*,*E*’), a phenotype that is consistent with an MT structural defect. Concurrently, others also showed that *spas-1* is required for TRN axon maintenance (Lee et al., 2025).

## Discussion

The molecular function of spastin in neurons has received significant attention since the description of its role in HSP (Errico et al., 2002). Here we show that *spas-1*, the *C. elegans* ortholog of spastin, is required for axon regeneration in GABA motor neurons and that *spas-1* functions cell autonomously in the neurons during regrowth after laser axotomy. Our results are aligned with earlier data in the *Drosophila* system, suggesting that the role of spastin in regrowth after injury is conserved across taxa, highlighting a need for MT severing and reorganization during regeneration (Stone et al., 2012; Rao et al., 2016). Axonal injury triggers several mechanisms, including MT reorganization in the injured axon (Kim and Jin, 2015). MT stress can activate the DLK-1 pathway and trigger cellular signaling involved in neuronal remodeling, MT growth, and axon regeneration (Hammarlund et al., 2009; Ghosh-Roy et al., 2012; El Bejjani and Hammarlund, 2012b; Chen et al., 2014). Importantly, the role of MT dynamics in axon regeneration is complex, with MT stabilizing drugs and MT capping positively impacting axon regeneration and MT-severing enzymes, like spastin, also being positively involved (Chen et al., 2011; Stone et al., 2012; Rao et al., 2016). Recent findings reconcile these apparently conflicting findings by identifying a mechanism by which spastin locally acts to promote MT growth during synapse formation and development, suggesting a potential recapitulation of this mechanism during axon regeneration (Aiken and Holzbaur, 2024).

Furthermore, we observed age-dependent progressive degeneration in the long anteroposterior axons of the TRNs in *C. elegans*, suggesting that spastin also functions in axonal maintenance in the worm as it does in flies and humans (Kammermeier et al., 2003; Sherwood et al., 2004; Du et al., 2010; Blackstone, 2012). Interestingly, the main age-related defect we observed in the *spas-1* null animals is an increase in a wavy appearance in the axons, suggesting a loss of internal structure that is consistent with a MT cytoskeletal defect.

Because of the central role that MTs play in structure, transport, and signaling in neurons, the dual role of spastin, a major MT-associated enzyme, in axon maintenance and regeneration is not surprising (Chen, 2018; Sferra et al., 2020). Importantly, other molecular mechanisms downstream of calcium signaling, a key injury response pathway, are dually implicated in axon regeneration and degeneration (Ding et al., 2022; Czech et al., 2023). More recently, it was shown that calcium signaling through CamKII stabilizes spastin in axons, hence linking calcium signaling to MT dynamics through spastin function in vertebrate neurons (Zou et al., 2025).

In summary, our data show for the first time that *spas-1* is required cell autonomously for axon regeneration in *C. elegans*, in accord with previous reports in *Drosophila*. Additionally, we show that mutant worms carrying a *spas-1* deletion display an age-dependent increased level of structural defects in axons, indicative of progressive axon degeneration in the worm system, as shown in mammals and flies. These findings may have implications for spastic paraplegia because our work and others show that spastin is involved in the maintenance of axons and regrowth after denervation.

## Synthesis

Reviewing Editor: Jennifer Dulin, Texas A&M University Decisions are customarily a result of the Reviewing Editor and the peer reviewers coming together and discussing their recommendations until a consensus is reached. When revisions are invited, a fact-based synthesis statement explaining their decision and outlining what is needed to prepare a revision will be listed below. The following reviewer(s) agreed to reveal their identity: NONE.

The reviewers agree that this manuscript contributes knowledge to the field of neural regeneration as it provides confirmation of results obtained in other species, and advances our knowledge on the function of spastin in regeneration and maintenance. The reviewers share concerns about interpretation of results, presentation of the data, and clarity of the analysis. Please see below.

-----

Reviewers' Comments:

Reviewer #1:

In the manuscript entitled "C. elegans spastin/spas-1 is required for axon regeneration and maintenance", the authors describe a novel role for spas-1, the C. elegans ortholog of spastin, in neuronal regeneration and maintenance, using the nematode as a model system. They provide evidence suggesting for the first time that spas-1 may be expressed in GABAergic motor neurons, promotes axon regeneration and appears to act cell-autonomously. Additionally, consistent with previous reports, they show that spas-1 is required for maintaining the morphology of TRNs. The manuscript is clearly written and contributes new insight into the conserved functions of spastin in neural regeneration and degeneration, with potential relevance to C. elegans biology and to the Hereditary Spastic Paraplegia research community.

However, I identified several major concerns that, in my opinion, warrant further experimental work essential to the report to substantiate the authors' claims and strengthen the manuscript's conclusions.

Major Concerns

1. Expression of spas-1 in TRNs

• The expression of spas-1 in TRNs has been previously reported (Brown and El Bejjani, microPublication 2017) using the same transcriptional reporter. This result is not shown in any figure in the present manuscript but is nonetheless claimed as a novel finding on page 4. I suggest the authors remove this statement and avoid presenting it as a new result.

2. Expression of spas-1 in GABAergic Motor Neurons

• The evidence for spas-1 expression in GABA neurons relies solely on transcriptional reporters, which are insufficient to definitively conclude expression in specific cell types (see https://doi.org/10.1093/genetics/iyae167 and https://doi.org/10.1895/wormbook.1.106.1). For conclusive evidence, a translational reporter or an endogenous tagged version of the gene should be used-such as the one described in Brown et al., microPublication 2024 (Luth Lab). This would validate the expression and support the rationale for the subsequent rescue experiments. Alternatively, the authors should tone down their conclusions regarding spas-1 expression in GABA neurons, clarify its limitations, and reference available literature for further support (e.g., CENGEN). Another option is to maintain the claim but ensure that rescue experiments in GABA neurons convincingly support it-which, at present, they do not (see below).

3. Confusion on the experiments made on TRN and GABA neurons.

• The manuscript lacks a consistent narrative when transitioning between data on TRNs and GABAergic neurons. It is often unclear whether the study focuses on one or both neuronal types, which weakens the logical flow. For example, at times the authors relate locomotion phenotypes to TRNs, which is questionable given their known sensory role and lack of direct impact on locomotion under unstimulated conditions.

• To strengthen the comparative perspective, I encourage the authors to explore whether spas-1 mutants exhibit age-related degeneration in GABA neurons or whether regeneration is similarly impaired in TRNs. Performing at least one of these experiments would add significant value.

• Furthermore, I recommend reorganizing the manuscript to follow a clear and linear structure, such as:

1. Physiological degeneration (age-dependent axon maintenance)

2. Insult derived degeneration/regeneration (axotomy)

3. Expression analysis

4. Tissue-specific rescue

4. Presentation of Regeneration Data (Figures 2C-E, 3A-B)

• Presenting regeneration results as raw numbers of "regenerated" vs. "failed" axons is problematic due to variable sample sizes across groups (N and n). I strongly recommend reporting regeneration as percentages (as in https://doi.org/10.1002/dvdy.22606 or https://doi.org/10.7554/eLife.12734).

• Additionally, the definition and consistency of the categories "regenerated" and "failed" are unclear, especially in Figures 2D and 3A-B, where discrepancies in the rescue trends raise concerns. In other words what happens in Fig. 2D to the many axons in spas-1; Pspas-1:spas1 not "failed", since the "regenerated" ones are only mildly increased? A clearer explanation of the scoring categories and the biological meaning of each class is needed, as well as a revision of the graphs using percentages to improve clarity and statistical interpretation.

5. Cell-Autonomous Rescue in GABAergic Neurons (Figure 3)

• Rescue experiments are currently based on only two extrachromosomal lines, and only one line in Figure 3 shows statistically significant rescue. This is insufficient to claim a robust cell-autonomous effect.

• It is standard in the C. elegans field to analyze at least three independent lines to ensure reproducibility, as expression levels can vary significantly between lines. Either another independent line should be added, or more animals should be scored per existing line B. Additionally, if data presentation is changed to percentages (see point 4 above), this may impact statistical outcomes.

• I also disagree with interpreting trends lacking statistical significance (especially with an N=17 or N=7) as meaningful (doi: 10.1093/bja/aev149). Thus, I believe the statements on page 14 claiming that "spas-1 expression in GABA motor neurons is sufficient to rescue regeneration" or "Genomic spas-1 driven by the unc-47 GABA motor neuron promoter, show increased regeneration in response to axotomy compared to spas-1(ok1608) in two separate extrachromosomal transgenic lines; increased regeneration is statistically significant in Line A." are currently not supported by the data and should be revised or removed

Minor Comments

• Page 6: Rephrase "Surprisingly, a deletion of the C. elegans spastin ortholog, spas-1, does not affect normal locomotion..." since this is only true at young stages; see doi: 10.1093/hmg/ddv632.

• Page 7: Similarly, rephrase "alleles move indistinguishably from wild type worms."

• Page 7: Clearly indicate in the text that axon breaks, branching from the soma, and wavy processes are shown in Figure 5B, 5C, and 5D, respectively.

• Page 12: Correct sentence starting with "To calculate the mean neuron defect per worm, The..."

• Page 12: Missing full reference for "(Toth et al.)"

• Page 16: Correct sentence "Crossed from RB1411 and REB127"

• Figure 2A: Should be cited in the text and include citation of WormBase as the source.

• Figure 2B: Add a further arrow on the right to indicate a third stump with "no regeneration."

• Figure 5E: The y-axis label should be simplified to "Neuron Defects Per Worm"; please quantify each single defect contributing to this metric.

• Table Expression clones (Page 16): Explain the meaning of the numbers used to describe expression clones.

• Nomenclature: Double-check strain names, also using WormBase or WormBook guidelines (e.g., assign codes to transgenic lines like Pspas-1::spas-1; Pspas-1::mCh; GABA::spas-1).

• Acknowledgments: Acknowledge CGC and WormBase as per their recommended policies.

Additional Points for Discussion

• The apparent spas-1 expression in cholinergic neurons (visible in Figure 1) should be acknowledged and discussed.

• Discuss the disease relevance of the findings, particularly in the context of spastin's role in hereditary spastic paraplegia.

• Justify the use of the ok1608 allele instead of tm683. Mention that ok1608 is a null allele (see 10.1093/hmg/ddv632), and reference studies where both alleles were used and produced similar results (e.g., 10.7554/eLife.102001.2 and 10.1093/hmg/ddv632).

• Consider whether the PLM branching phenotype in mutants is replacing the normal anterior branching pattern.

Reviewer #2:

In this study entitled "C. elegans spastin/spas-1 is required for axon regeneration and

maintenance" the authors identify a function of Spastin 1 in regulating axonal regeneration and axonal maintenance in a subset of C. elegans neurons, confirming similar data obtained previously in Drosophila. Using fluorescent tagging, the authors reveal that SPAS-1 is expressed in GABAergic motor neurons (and they state in the mechanoreceptor neurons, but this result is not shown). They show that general development of these neurons is not altered by the spas-1 mutation, whereas upon laser-induced axonal injury, the spas-1 mutation impacts the capacity of the GABAergic neurons to mount an axonal regenerative response. Using cell-specific promoters, they demonstrate that the function of SPAS-1 in this axonal regenerative context is cell-autonomous. Finally, they reveal that SPAS-1 also impacts the maintenance of the axons, which appear to lose integrity as the animal age. The paper covers some interesting concepts and provides confirmation of results obtained in the fruit fly, as clearly stated by the authors. However, it will need to be highly improved in several aspects such as structure, presentation, and style to ensure it fits the standard of this or other journals and be further considered.

Main conceptual elements.

1. The paper should include as the first figure a photo and a scheme of the GABAergic neurons and mec neurons, to ensure the reader can understand the model (similar to Figure 4 but coupled with a schematic diagram of the neurons).

2. Given that the authors use the same transgenic strains to visualise neurons, it might be simpler to rearrange the paper as follows: i) spas-1 is expressed in GABA motor neurons and in TRNs, ii) spas-1 is not required for initial nervous system development. iii) spas-1 is required for axon regeneration in GABA motor neurons iv) spas-1 functions cell-autonomously to regulate axonal regeneration v) spas-1 is required for maintenance of the mec neurons.

3. Although the authors claim that spas-1 is expressed in the touch-receptor neurons (TRN), this is not shown in the results. This is referenced in the text as being Fig. 1, but in this panel, only GABAergic motorneurons are shown. Please provide the data for this claim.

4. Current quantification of regeneration is unconventional and difficult to compare with literature data or within other results of this paper. This would be better expressed as a percentage of neurons that initiated regeneration. Without this information it is difficult to conclude any effect, and it is difficult to interpret what sample size has been used to determine statistical significance of the results. Which one was used? The number of animals or the number of axons? Finally, with the data expressed in percentage, the two rescued strains generated could be on the same bar graph.

5. Figure 3. As above, the tissue specific rescue of the axonal regeneration phenotype should be expressed as percentage, and both strains present in the same graph.

6. The authors communicate that spas-1 does not affect neuronal development given the absence of an abnormal locomotion or neuronal structure phenotype. It is difficult to conclude this without any meaningful quantification or clear representative images of both neurons, or animal locomotion. How many animals were observed for each of these analyses? Moreover, it seems there is a contradiction in the text where the authors first state there is no major axon guidance or neuronal structural phenotypes, but then a few paragraphs later they state that there are TRN phenotypes regardless of age. This phenotype should be quantified.

7. In Fig. 5 the authors mention that spas-1 affect TRN maintenance, but how? It would be interesting to narrow down which aspect of maintenance it is controlling. Is it structural integrity? Guidance? Elasticity? This can be answered by simply quantifying and represent individually each of the observed defects.

Minor points:

8. There are many inconsistencies in the nomenclature of transgenes. Please use the convention for the field. For example, instead of 'GABA:spas-1' use 'GABA::spas-1'.

9. Furthermore, the unc-47 promoter is referred to using GABA, pGABA, unc-47. Please use one term throughout the text.

10. Abbreviations should be introduced in full before their first appearance. The abbreviation TRN is never fully introduced

11. It is difficult to follow which transgenes are used along the paper. Please refer to the transgene using their name, i.e. axrEx58...

12. No reference to Fig. 2A in the text.

13. Panels in Fig. 4 should be separated and be the same size, so that is clear which image belongs to each genotype. As indicated above, the wild type animals of both transgenic strains could be used as Fig. 1 each accompanied with its own individual schematic

14. Fig. 5 should be properly annotated to describe the defects the authors are referring to in the text. Fig. 5E sample size is not readable, please reformat it. In the same plot, the column labels are confusing. Perhaps plotting in separate graphs would help readability.

15. On the methods section, in the 'Axon maintenance/degeneration scoring' subsection there is a broken reference in the following: "...each experimental group as described (Toth et al.)."

16. Page 3, "Drosophila" should be in italics.

17. Page 3 two lines from the bottom, "maintenance in worms are not described" replace with: "maintenance in this species are not characterized". Last line of the page, add comma after spas-1.

18. Page 4, fourth line of the Results: " We crossed an extrachromosomal array transgenic line expressing.." replace with: "We crossed a transgenic line expressing.."

19. Page 4, second line cell autonomously should have a hyphen.

20. Page 5, "we crossed the spas-1(ok1608)" replace with: "we genetically crossed the spas-1(ok1608)"

21. Page 5, line 8: "as evidence of cutting", replace with: "as evidence of successful axotomy".

22. Page 5, line 9: "regeneration past the cut site in C. elegans", replace with: "regeneration in C. elegans".

23. Page 5, line 9: "..driven by the same promoter we previously used.." replace with: " driven by the endogenous promoter previously used.."

24. Page 5, line 12: "...seen in wild type worms in two independent transgenic lines (Fig. 2D and E)." replace with: "...seen in wild type animals (Fig. 2D and E)."

25. Page 5, subtitle: "Expression of spas-1 in GABA motor neurons is sufficient for the rescue of axon regeneration." replace with: "spas-1 functions cell-autonomously in GABA motor neurons to regulate axon regeneration.

26. Page 5, last sentence "Axon regeneration is significantly improved in one transgenic line and is trending towards rescue in a second line, suggesting that spas-1 functions cell autonomously in GABA motor neurons as a positive regulator of axon regeneration." replace with: "Thus, spas-1 functions cell autonomously in GABA motor neurons as a positive regulator of axon regeneration".
